# Dried fruit pomace inclusion in poultry diet: growth performance, intestinal morphology and physiology

**DOI:** 10.1186/s40104-020-00464-z

**Published:** 2020-06-19

**Authors:** Elena Colombino, Ilario Ferrocino, Ilaria Biasato, Luca Simone Cocolin, Daniel Prieto-Botella, Zenon Zduńczyk, Jan Jankowski, Joanna Milala, Monika Kosmala, Bartosz Fotschki, Maria Teresa Capucchio, Jerzy Juśkiewicz

**Affiliations:** 1 Department of Veterinary Sciences, University of Torino, Largo Paolo Braccini 2, Grugliasco, 10095 Turin, Italy; 2 Department of Agricultural, Forest and Food Sciences, University of Torino, Torino, Italy; 3 Research Team on Occupational Therapy (InTeO), Department of Surgery and Pathology, University Miguel Hernandez of Elche, Alicante, Spain; 4 Polish Academy of Sciences, Institute of Animal Reproduction and Food Research, Olsztyn, Poland; 5 Department of Poultry Science, University of Warmia and Mazury, Olsztyn, Poland; 6 Institute of Chemical Technology of Food, Lodz University of Technology, Lodz, Poland

**Keywords:** Fruit Pomace, Gut health, Morphohistology, Polyphenols, Poultry

## Abstract

**Background:**

Fruit pomaces are by-products rich in polyphenol compounds and dietary fiber. They seem to play an important role in regulating the gut microbiota, morphology and physiology. The aim of this study was to assess whether apple (A), blackurrant (B) or strawberry (S) pomaces could be suitable ingredients in broiler diets and their effect on gut health. A total of 480 male broilers were randomly allotted to 8 dietary treatments with lower (3%-L) or higher (6%-H) dietary fiber content: two control groups (CL/CH), two A diets (AL/AH), two B diets (BL/BH), two S diets (SL/SH). Diet and fruit pomaces were chemically analyzed to assess polyphenol concentration and fibre fraction content. After the evaluation of growth performance, 6 birds/group were slaughtered at 35 days of age. Morphometric and histopathological investigations were performed on duodenum, jejunum and ileum. Excreta were collected to perform microbiota evaluation by 16S DNA sequencing. Weight, viscosity, enzymatic activity, short chain fatty acid (SCFAs) and ammonia concentration were determined in ileum and/or ceca content.

**Results:**

A pomace and A diets showed the lowest polyphenol content and the highest content of soluble fibre fraction. No significant differences were observed for growth performance, gut morphometry and histopathology (*P* > 0.05). Dietary fruit pomace inclusion increased the weight of ileum and ceca and the ileum digesta viscosity (*P* < 0.05). In the ileum, A and S groups showed lower bacterial α-glucosidase activity than C groups. Moreover, small intestine SCFAs concentration was higher in fruit pomaces diets (*P* < 0.05). In ceca, B and S groups showed lower ammonia concentration and higher SCFAs than C. Dietary treatments also influenced the activity of α-glucosidase, α-galactosidase, β-galactosidase β-glucuronidase and xylase. Regarding microbiota, at phylum level, Firmicutes were differentially abundant across treatment (maximum for C and minimum in S, FDR > 0.05). At genus level, an increase of *Weissella* in AH and *Erwinia* in S/B diets, as well as a decrease of *Lactobacillus* in all fruit pomace groups were recorded (*P* < 0.05).

**Conclusions:**

Fruit pomaces could be suitable ingredients in poultry nutrition even if further studies are needed to better understand which doses is more recommended to avoid negative effects on gut microbiota.

## Introduction

Fruit juice production resulted in a considerable amount of by-products that are rich sources of bioactive compounds [1]. Researches demonstrated that fruit pomaces are rich in phenolic compounds, particularly seeds and peel [2–4]. It is well known that polyphenols seemed to be responsible for many health benefits often associated with a lower risk of chronic diseases [5–7]. Moreover, the antioxidant compounds in fruit pomaces could be used for increasing the stability of foods by preventing lipid peroxidation and oxidative damage in living systems by scavenging oxygen free radicals [8]. To reduce production waste, these by-products can be utilized as a novel, low-cost, and natural sources of dietary fiber and antioxidants in poultry nutrition. In fact, dried fruit pomaces contain around 50–70% of dietary fibers, and they are rich source of polyphenols [9, 10]. On one hand, high contents of dietary polyphenol in chicken diets (2.5 g/kg or more) might reduce ileal protein digestibility and weight gain, with a negative influence on feed conversion in birds [11]. On the other hand, balanced diet with increased level of polyphenol can prevent lipid oxidation in broiler diets that contain high levels of unsaturated fatty acids [12]. Furthermore, polyphenolic profile in diet may also play a significant role in regulating the microbial activity of the gastrointestinal tract, and thus the physiological morphology and functionality of intestine [13]. Recently, different type of fruit pomaces have been studied as a novel food in poultry nutrition. In particular, some studies showed that dried fruit pomaces from berries added to diets increased oxidative stability of the turkey meat and did not impair the growth performances [10, 14]. Furthermore, Goni et al., [12] stated that grape pomaces can be effectively incorporated into maize-soybean basal diets for broiler chickens. Akhlaghi et al., [15] also performed nutritional studies on roosters and concluded that long-term administration of dried apple pomace improved their reproductive performances. However, nowadays fruit pomaces are used in poultry nutrition only locally, mostly in developing countries, to reduce feed costs [16]. There is still little knowledge on how the dosage and the type of fruit pomace may affect the growth performances, feed utilization and physiology of broilers. Therefore, the aim of this study was to evaluate the effects of dried fruit pomaces (apple, blackcurrant and strawberry pomaces) inclusion on growth performance, intestinal morphology and physiology of broiler chickens.

## Materials and methods

### Birds and husbandry

The trial was carried out at the Research Laboratory of the Department of Poultry Science, University of Warmia and Mazury in Olsztyn (Poland) in cooperation with the University of Torino and the Institute of Animal Reproduction and Food Research of PAS in Olsztyn. The experimental protocol was approved by the Local Animal Care and Use Committee (Decision No. 2/2018; Olsztyn, Poland), and the study was carried out in accordance with EU Directive 2010/63/EU for animal experiments. The temperature and lighting program were consistent with the recommendations of Aviagen Group [17]. The birds had free access to feed and water. A total of 480 Ross 308 male broilers at one-day of age were randomly allotted to 8 dietary treatments, each consisting of 6 pens as replicates with 10 birds per pen. The one-day-old chickens were purchased from a commercial hatchery (Animex Group, Sokolka, Poland).

### Diets

A control diet (C) based on wheat, corn meal and soybean meal was formulated and Vitacel® cellulose (Rettenmaier, Warsaw, Poland) preparation was added as fibre component. Apple pomace (A), blackcurrant pomace (B) and strawberry pomace (S), dried in the SB-1.5 rotary drum dryer for biomass residues, were supplied by Agro-Bio-Produkt Sp. z o.o. in Grodkowice (Poland), and were added to the experimental diets at 3% (L, low cellulose) or 6% (H, high cellulose) inclusion levels as a fibre source. Crude fibre content was estimated at 3.2–3.3% (L) and 3.9–4.0% (H) in all the diets, respectively. For each dietary treatment, diets were divided into two phases: a starter diet (1–14 days) and a grower/finisher diet (15–35 days) (Table 1). The nutritional value of the experimental diets met the broiler nutrient requirements [18].

**Table 1:** Composition of diets fed to broilers at starter and grower period, %

	Starter diets (days 1–14)								Grower diets (days 15–35)							
	CL	CH	AL	AH	BL	BH	SL	SH	CL	CH	AL	AH	BL	BH	SL	SH
Wheat	43.0	43.0	37.9	32.9	39.2	35.5	39.3	35.5	47.0	46.9	42.0	36.9	43.3	39.6	43.3	39.6
Corn	20.0	20.0	20.0	20.0	20.0	20.0	20.0	20.0	20.0	20.0	20.0	20.0	20.0	20.0	20.0	20.0
Soybean meal	29.6	29.6	30.5	31.3	29.5	29.4	29.5	29.3	24.5	24.5	25.3	26.1	24.4	24.3	24.3	24.1
Cellulose	0.70	1.38	–	–	–	–	–	–	0.70	1.38	–	–	–	–	–	–
Apple pomace	–	–	3.00	6.00	–	–	–	–	–	–	3.00	6.00	–	–	–	–
Blackcurrant pomace	–	–	–	–	3.00	6.00	–	–	–	–	–	–	3.00	6.00	–	–
Strawberry pomace	–	–	–	–	–	–	3.00	6.00	–	–	–	–	–	–	3.00	6.00
Soybean oil	2.83	2.83	4.02	5.22	3.58	4.33	3.62	4,42	4.47	4.52	5.67	6.86	5.23	5.98	5.27	6.07
Sodium bicarbonate	0.15	0.15	0.15	0.15	0.15	0.15	0.15	0.15	0.15	0.15	0.15	0.15	0.15	0.15	0.15	0.15
Fodder salt	0.23	0.23	0.23	0.23	0.23	0.23	0.23	0.23	0.23	0.23	0.23	0.23	0.23	0.23	0.23	0.23
Limestone	1.29	1.29	1.27	1.25	1.28	1.27	1.28	1.27	1.09	1.02	1.08	1.06	1.08	1.07	1.09	1.08
Mon-Ca phosphate	0.87	1.19	1.60	1.64	1.60	1.64	1.60	1.64	0.53	0.53	1.26	1.29	1.26	1.29	1.26	1.29
*DL*-Methionine 99	0.37	0.37	0.38	0.39	0.39	0.41	0.39	0.41	0.34	0.34	0.35	0.36	0.36	0.38	0.36	0.38
*L*- Lysine 99	0.33	0.33	0.33	0.32	0.35	0.38	0.36	0.38	0.35	0.35	0.34	0.33	0.37	0.39	0.37	0.39
*L*-Threonine	0.15	0.15	0.15	0.15	0.16	0.18	0.16	0.18	0.16	0.18	0.16	0.16	0.17	0.19	0.17	0.19
Vit-min premix^a^	0.50	0.50	0.50	0.50	0.50	0.50	0.50	0.50	0.50	0.50	0.50	0.50	0.50	0.50	0.50	0.50

*CL* Control diet with 3% of cellulose; *CH* control diet with 6% of cellulose; *AL* 3% inclusion level of apple pomace; *AH* 6% inclusion level of apple pomace; *BL* 3% inclusion level of blackcurrant pomace; *BH* 6% inclusion level of blackcurrant pomace; *SL* 3% inclusion level of strawberry pomace; *SH* 6% inclusion level of strawberry pomace

^a^Contents per kg feed: vitamin A, 12,500 IU; vitamin D_3_, 3,500 IU; vitamin E, 50 mg; vitamin K_3_, 3 mg; vitamin B_1_, 3 mg; vitamin B_2_, 8 mg; vitamin B_6_, 5 mg; vitamin B_12_, 0.025 mg; biotin, 0.25 mg; calcium pantothenate, 12 mg; nicotinic acid, 50 mg; folic acid, 2 mg; choline chloride, 400 mg; Fe, 50 mg; Mn, 100 mg; Zn, 100 mg; Cu, 12 mg; I, 1 mg; Se, 0.3 mg

### Chemical analyses of pomaces and experimental diets

Fruit pomaces were analysed to establish the dry matter, crude protein, crude fat, crude fibre, crude ash, total dietary fibre and soluble and insoluble fibre fraction (TDF, SDF, IDF, respectively) according to AOAC International [19] [see Additional file 1]. Selected macroelements (Ca, K, P, Mg, Na) were also determined for apple, blackcurrant and strawberry pomaces. The samples were mineralized in a mixture (3:1) of nitric and perchloric acids (Merck, Darmstadt, Germany) to determine the mineral composition of pomaces. Weighed samples were mineralized in a VELP DK 20 electric aluminium heating block with selectable temperatures (VELP Scientifica, Usmate Velate, Italy). The Ca, K, Mg and Na contents of mineralized samples were determined by flame atomic absorption spectrometry (acetylene-air flame). The analysis was performed using a Unicam 939 Solar atomic absorption spectrophotometer equipped with an Optimus data station, background correction system (deuterium lamp) and cathode lamps. The P content of mineralized samples was determined by colourimetry using ammonium molybdate, Na sulphate and hydroquinone. Absorbance was measured in a VIS 6000 spectrophotometer (Krüss–Optronic, Hamburg, Germany) at a wavelength of λ = 610 mm. In the pomaces and in the experimental diets the polyphenolic fraction and TDF, SDF, IDF were analysed. The content of the nutrients and non-nutrients in the diets was calculated according to the Polish Feedstuff Analysis Tables [20] and the analysed fruit pomaces.

High-performance liquid chromatography diode array detector (HPLC DAD) was used for the determination of polyphenolic content in fruit pomaces and experimental diets. Analysis of polyphenols by HPLC DAD was performed by using a Smartline chromatograph (Knauer, Berlin, Germany) with a PDA detector and a Gemini 110A 5 μm C18 column (250 mm× 4.60 mm) (Phenomenex, Torrance, USA) at 35 °C with a flow rate 1.25 mL/min. Phase A was phosphoric acid in water (0.05/99.95, v/v), and phase B was phosphoric acid, acetonitrile, and water (0.05/83/16.95, v/v). The gradient was as follows: 0–5 min, 4% B; 5–12.5 min, 4–15% B; 12.5–42.5 min, 15–40% B; 42.5–51.8 min, 40–50% B; 51.8–53.4 min, 50–55% B; and 53.4–55 min, 4% B. Detection was at 250 nm (ellagitannins), 360 nm (ellagic acid, quercetin, and kaempferol glycosides as well as aglycons, phloridzin), and 520 nm (anthocyanins). The standards applied were cyanidin-3-O-glucoside, pelargonidin-3-O-glucoside ellagic acid, kaempferol, kaempferol-3-O-glucoside, quercetin, and quercetin-3-O-glucoside, phloridzin, chlorogenic acid tiliroside (Extrasynthese, Genay, France). Agrimoniin standard was obtained as described by Sójka et al. [21]. The total polyphenol content was determined spectrophotometrically according to Singleton and Rossi [22]. Procyanidins were determined by excess phloroglucinol degradation method according to Kennedy and Jones [23] .

### Growth performance

The trial lasted 35 days. The body weight (BW) and the body weight gain (BWG) were recorded on days 14 and 35. Mortality rate was monitored daily. Daily feed intake (DFI) and feed conversion ratio (FCR) were calculated for the whole experimental period (1–35 days).

### Pre-slaughter procedures

At day 34, all birds were individually weighed and 12 birds/diet were selected on the basis of pen average live weight, tagged and fasted for 8 h. The birds were electrically stunned (400 mA, 350 Hz), hung on a shackle line and exsanguinated by a unilateral neck cut severing the right carotid artery and jugular vein.

### Histomorphological investigations

Six birds per feeding group were submitted to intestinal morphometric investigations and histopathological evaluation. Samples of duodenum, jejunum and ileum were excised and flushed with 0.9% saline to remove all the content. The collected segments of intestine were the loop of duodenum, the tract before Meckel’s diverticulum (jejunum) and the tract before the ileocolic junction (ileum). Gut segments were fixed in 10% buffered formalin solution for morphometric analysis. Tissues were routinely embedded in paraffin wax blocks, sectioned at 5 μm thickness, mounted on glass slides and stained with Haematoxylin & Eosin (HE). The evaluated morphometric indices were villus height (Vh, from the tip of the villus to the crypt), crypt depth (Cd, from the basis of the villus to the submucosa), and the villus height to crypt depth (Vh/Cd) ratio, [24]. Morphometric analyses were performed on 10 well-oriented and intact villi and 10 crypts chosen from duodenum, jejunum, and ileum [25]. The observed histopathological findings were evaluated using a semi-quantitative scoring system as follows: absent (score = 0), mild (score = 1), moderate (score = 2) and severe (score = 3). Gut histopathological findings were separately assessed for mucosa (inflammatory infiltrates) and submucosa (inflammatory infiltrates and Gut-Associated Lymphoid Tissue [GALT] activation) for each segment. The total score of each gut segment was obtained by adding up the mucosa and submucosa scores.

### Small intestinal and cecal physiological indices

Six birds per group were submitted to intestinal physiological measurements (small intestine-SI and ceca-Ce). As soon as possible after euthanasia (ca. 10 min), pH was measured directly in the ileum and the ceca using a microelectrode and a pH-ion meter (model 301, Hanna Instruments, Vila do Conde, Portugal). Samples of ileal (middle 1/3 section of the segment) content were collected for the analysis of viscosity, enzymatic activity and short-chain fatty acids (SCFAs) content. Pooled samples of ileal digesta were vortexed and centrifuged at 7,211×*g* for 10 min. The supernatant fraction (0.5 mL) was placed in the Brookfield LVDV-II+ cone-plate rotational viscometer (CP40; Brookfield Engineering Laboratories, Stoughton, MA, USA), and the viscosity of pooled samples was measured at a constant temperature of 39 °C and a shear rate of 60 s^− 1^. Viscosity was recorded as apparent viscosity. Fresh samples of the cecal contents were used for the analysis of dry matter (DM), ammonia and enzymatic activity. The DM content of cecal digesta was determined at 105 °C. Ammonia was extracted from fresh cecal digesta, trapped in a solution of boric acid in Conway dishes and determined by direct titration with sulphuric acid [26].

The SCFA concentration in ileal and cecal digesta was analysed by gas chromatography (GC) (Shimadzu GC-2010, Kyoto, Japan). The detailed description of SCFA GC analyses methods were provided previously in Zduńczyk et al. [27]. The concentration of ileal/cecal putrefactive SCFA (PSCFA) were calculated as the sum of iso-butyrate, iso-valerate and valerate in digesta. All SCFA analyses were performed in duplicate. Pure acetic, propionic, butyric, iso-butyric, iso-valeric and valeric acids were obtained from Sigma (Poznan, Poland), and they were combined to create a standard plot and calculate the amount of each acid. The additional set of pure acids was included in each GC run at five sampling intervals to maintain calibration. The activity of jejunal mucosal maltase was assayed as described by Opyd et al. [28]. The activity of bacterial enzyme α-glucosidase in the ileal digesta as well as activities of cecal bacterial enzymes (α-glucosidase, α- and β-galactosidase, β-glucuronidase, α-arabinofuranosidase, and β-xylosidase) were measured by the rate of release of *p*-nitrophenol or *o*-nitrophenol from the respective nitrophenylglucosides, according to Grzelak-Blaszczyk et al. [29]. Enzyme activity was expressed in μmol of the product formed per hour per gram of ileal digesta or per gram of protein in the cecal digesta. Protein content of the cecal digesta was determined by the Lowry’s method using the Folin phenol reagent and bovine serum albumin as standard. All analyses were performed in duplicate. After digesta sampling, the small intestine and the ceca was flushed with water, blotted on filter paper and weighed.

### Excreta microbiota characterization

At the end of the trial, in order to collect excreta samples, all the birds were removed from each pen and housed in wire-mesh cages (100 cm width × 50 cm length) for 120 min to collect fresh excreta samples in order to obtain six replicates/treatment. Samples were pooled (by pen/replicate) and then transferred with a sterile spatula in an eppendorf tube to be stored at − 80 °C prior DNA extraction and sequencing. Total DNA was extracted from the samples using the RNeasy Power Microbiome KIT (Qiagen, Milan, Italy) following the manufacturer’s instructions. One microliters of RNase (Illumina Inc, San Diego, CA, USA) was added to digest RNA in the DNA samples with an incubation of 1 h at 37 °C. DNA directly extract from excreta samples was used to assess the microbiota by the amplification of the V3-V4 region of the 16S rRNA gene using primers and protocols according to the 16S metagenomic sequencing library preparation instructions. The paired-end sequencing reaction (2 × 250 bp) was performed using the Illumina MiSeq platform according to the manufacturer’s instructions.

### Bioinformatics and statistical analysis

The data were analysed using Statistica 12.0 Software and IBM SPSS Statistics V25.0.0 software. Individual birds were considered as experimental units to analyse gut morphology and intestinal parameters while each pen was considered as experimental unit for growth performances. The Shapiro–Wilk test was used to test the normality of the data distribution before statistical analyses. Data were described by mean and standard deviation (SD) or median and interquartile range (IR) depending on data distribution. Bivariate analysis was performed by Chi-square, Kruskall Wallis and one-way ANOVA test. Variables that shown significant differences in bivariate analysis were analysed by two-way ANOVA (IBM SPSS General Linear Model-Univariata). The model allowed the evaluated variables to depend on three fixed factors (diet, cellulose level, and interaction between diet and cellulose level). The interactions between the levels of the fixed factors were evaluated by pairwise comparisons (Tukey Test). In order to explore the magnitude and attitude of the relationship between diet and the most relevant gut parameters (SI weight, SI viscosity, SI SCFA, SI PSCFA, SI butyric acid, Ce weight, Ce SCFA, Ce PSCFA and Ce butyric acid), linear regression was performed using the following equation: Y = β_0_ + β_1_X_1_ + β_2_X_2_ + β_3_X_3_ + β_4_X_4._ where Y = dependent variables, β_1_X_1_, β_2_X_2_, β_3_X_3_ = dummy variables of diets (A, B, S), β_4_X_4._ = cellulose level. *P* values < 0.05 were considered statistically significant.

For microbiota analysis, paired-end reads were first merged using FLASH software [30] with default parameters. Joint reads were further quality filtered (at Phred < Q20) using QIIME 1.9.0 software [31] and the pipeline recently described [32]. A filtered OTU table was generated at 0.1% abundance in at least 2 samples through QIIME. Alpha diversity indices were calculated using the diversity function of the vegan package [33]. OTU table and alpha diversity index were used to find differences as a function of the dietary inclusion by using the Wilcoxon rank sum test. A Generalized Linear Model was used in order to test the importance of continuous or discrete variables available for the birds (sampling time and diet) on the relative abundance of bacterial genera or family.

## Results

### Chemical analyses of pomaces and experimental diets

The fruit pomaces used as feed components in the present trial differed in the content and composition of nutrients and non-nutrients, including fibre and polyphenolic fractions [see Additional file 1]. In comparison with the other pomaces, the A pomace was characterized by a lower content of crude protein and crude fat and relatively low concentrations of crude ash. In comparison with A pomace, crude fibre levels were lower in B and higher in S pomace. Total dietary fibre (TDF) content was comparable in the A and B pomaces (60.8% and 60.6%, respectively), while the S pomace showed lower TDF content (52.8%) than A and B diets. Moreover, A and B pomaces contained 9.1% and 7.7% of soluble fibre fraction (SDF), whereas S contained only traces of this fibre fraction (0.40%). The insoluble dietary fibre fraction (IDF) content was similar in all the pomaces (51.7–52.9%). In all the analysed fruit pomaces, procyanidins were the dominant polyphenolic fraction. The lowest concentration of polyphenols was noted in the A pomace (8.43 mg/g in HPLC analysis), which contained procyanidins (6.75 mg/g) and small amounts of chlorogenic acid (0.26 mg/g), quercetin glucosides (0.83 mg/g) and phloridzin (0.55 mg/g). The B pomace was characterized by more than 3-fold higher polyphenol content (26.7 mg/g) in comparison with the A groups, also containing anthocyanins (3.74 mg/g) and myricetin glycosides (0.34 mg/g) in addition to procyanidins (22.5 mg/g). The highest concentration of polyphenols was attributed to the S pomace (28.9 mg/g). The polyphenolic fraction of strawberry pomace was composed of procyanidins (15.8 mg/g), ellagitannins (11.2 mg/g) and small content of tiliroside, ellagic acid, quercetin glycosides and anthocyanins (0.85, 0.57, 0.27, and 0.14 mg/g, respectively).

The above-mentioned differences in the polyphenolic compounds in fruit pomaces also influenced the analysed content of total polyphenols and procyanidins in the experimental diets fed to broilers during the starter and grower-finisher feeding periods [see Additional file 2]. In comparison with the control diet, as observed for apple pomaces, the lowest increase in polyphenol levels was noted in AL and AH diets.

### Growth performance

Growth performances of the broiler chickens were summarized in Additional file 3. Dietary inclusion of fruit pomaces did not significantly influence the growth performance (*P* > 0.05). The mortality rates were low in all groups (0.0–3.7%) and were not affected by dietary treatments (*P* > 0.05).

### Histomorphological investigations

Data regarding histomorphological investigations are reported in Table 2. No statistically significant differences were recorded for Vh and Cd in duodenum, jejunum and ileum (*P* > 0.05). However, duodenum and jejunum showed higher Vh and Vh/Cd when compared to ileum. Histopathological alterations developed in all the intestinal segments, for all the dietary treatments. Occasional, mild to moderate lymphoplasmacytic infiltrates and lymphoid tissue hyperplasia were observed in duodenum, jejunum, ileum and ceca. Dietary apple, blackcurrant and strawberry pomace inclusion did not affect the severity of the observed histopathological alterations (*P* > 0.05).

**Table 2:** Histomorphological evaluation of small intestine and ceca of the broiler chickens

	Diet groups								
General variables	CL	CH	AL	AH	BL	BH	SL	SH	*P*-value
Villus height, mm									
Duodenum*	3.2 (0.13)	2.9 (0.4)	2.6 (0.4)	2.9 (0.6)	3.1 (0.4)	3.1 (0.4)	3.0 (0.5)	2.5 (0.4)	0.068
Jejunum*	2.1 (0.5)	1.8 (0.5)	2.1 (0.4)	1.8 (0.4)	1.9 (.03)	1.7 (0.3)	1.9 (0.6)	1.9 (0.4)	0.774
Ileum**	1.24 (1.0–1.5)	1 (0.8–1.0)	1.3 (1.1–1.4)	1.2 (1.1–1.4)	0.9 (0.8–0.9)	1.1 (1.0–1.4)	1.1 (0.8–1.6)	1 (0.9–1.1)	0.056
Crypth depth, mm									
Duodenum*	0.19 (0.01)	0.2 (0.01)	0.19 (0.03)	0.23 (0.03)	0.19 (0.05)	0.21 (0.03)	0.21 (0.04)	0.18 (0.04)	0.378
Jejunum**	0.19 (0.17–0.21)	0.16 (0.14–0.19)	0.22 (0.21–0.25)	0.21 (0.19–0.22)	0.22 (0.21–0.25)	0.19 (0.15–0.21)	0.19 (0.17–0.22)	0.18 (0.17–0.19)	0.197
Ileum**	0.17 (0.15–0.18)	0.17 (0.16–0.19)	0.17 (0.16–0.2)	0.18 (0.16–0.2)	0.19 (0.18–0.2)	0.17 (0.17–0.2)	0.18 (0.17–0.21)	0.17 (0.15–0.19)	0.703
Villus height/crypt depth ratio, mm/mm									
Duodenum*	16.3 (1.2)	14.5 (1.8)	13.4 (3.2)	12.5 (2.7)	16.7 (4.5)	14.7 (2.4)	14.4 (2.5)	13.9 (2.1)	0.159
Jejunum**	10.9 (8.9–13)	10.3 (10–10.6)	10.2 (9.5–10.4)	8.3 (7.0–9.6)	8.7 (7.6–10.1)	8 (8.0–8.1)	9.8 (8.4–10.9)	9.9 (9.4–10.3)	0.177
Ileum**	7.5 (6.1–8.6)	5.2 (4.8–5.8)	7.3 (5.8–8.3)	6.5 (6–8.7)	4.8 (4.7–4.9)	5.9 (5.7–6.9)	5.9 (5.4–6.8)	6.1 (4.4–6.6)	0.022
Duodenum inflammation, *n* (%)									0.097
Normal	2 (33.3)	2 (33.3)	2 (33.3)	6 (100)	2 (33.3)	2 (33.3)	1 (16.7)	4 (66.7)	
Mild-Moderate	4 (66.7)	4 (66.7)	4 (66.7)	0 (0)	4 (66.7)	4 (66.7)	5 (83.3)	2 (33.3)	
Jejunum inflammation, *n* (%)									0.548
Normal	4 (66.7)	1 (16.7)	2 (33.3)	3 (50)	4 (66.7)	4 (66.7)	3 (50)	4 (66.7)	
Mild-Moderate	2 (33.3)	5 (83.3)	4 (66.7)	3 (50)	2 (33.3)	2 (33.3)	3 (50)	2 (33.3)	
Ileum inflammation, *n* (%)									0.619
Normal	4 (66.7)	3 (50)	1 (16.7)	4 (66.7)	2 (33.3)	2 (33.3)	3 (50)	2 (33.3)	
Mild-Moderate	2 (33.3)	3 (50)	5 (83.3)	2 (33.3)	4 (66.7)	4 (66.7)	3 (50)	4 (66.7)	
Ceca inflammation, *n* (%)									0.120
Normal	4 (66.7)	3 (50)	6 (100)	3 (50)	4 (66.7)	6 (100)	6 (100)	5 (83.3)	
Mild-Moderate	2 (33.3)	3 (50)	0 (0)	3 (50)	2 (33.3)	0 (0)	0 (0)	1 (16.7)	

*CL* Control diet with 3% of cellulose; *CH* control diet with 6% of cellulose; *AL* 3% inclusion level of apple pomace; *AH* 6% inclusion level of apple pomace; *BL* 3% inclusion level of blackcurrant pomace; *BH* 6% inclusion level of blackcurrant pomace; *SL* 3% inclusion level of strawberry pomace; *SH* 6% inclusion level of strawberry pomace. *SD* standard deviation; *IR* interquartile range. *mean (SD), **median (RI)

### Small intestinal parameters

The evaluated small intestinal parameters were summarized in Table 3. The effect of diet, cellulose level and interaction between them over the small intestine variables are reported in Table 4. In order to explore the magnitude and attitude of the relationship between diet and the evaluated parameters, linear regression results are reported in Table 5. SI weight means showed significant differences among the dietary treatments (*P* < 0.001). In particular, it depends on diet (*P* < 0.001) and cellulose level (*P* = 0.03): C groups presented a lower SI weight when compared to A, B and S diets with the weight being also greater in the H diet than in the L diet. Linear regression showed that the higher increase in term of SI weight was given by A diet (*P* < 0.001). In fact, changing form C to A diet would increase small intestine weight of 5.863 g/kg BW. Moreover, SI weight would increase of 1.871 g/kg BW when L diet is replaced by H diet (*P* = 0.042). SI digesta viscosity significantly differed among the groups and it was influenced only by diet (*P* < 0.001)*.* In particular, C, B and S diets showed a lower viscosity when compared to A diets (*P* = 0.003). Linear regression showed that changing from C to A diet would increase viscosity of 0.528 mPa·s (*P* < 0.0001). Moreover, also SCFAs and PSCFAs concentration was influenced by diet (*P* = 0.001) and by interaction between diet and cellulose level (SCFAs only, *P* = 0.01) with a higher amount of SCFA in A, B and S diets when compared to C groups and a lower amount of PSCFAs in B and S diet than in A and C diets (*P <* 0.001). In particular, acetic and butyric acid concentration was higher in the small intestine of fruit pomace dietary treatments than in C diets (*P* < 0.001). Linear regression showed that replacing C diet with S diet would increase SCFAs of 1.37 μmol/g of digesta and decrease PSCFAs of 0.052 μmol/g of digesta, followed by B diet (0.657 μmol SCFAs more/g of digesta and 0.057 μmol PSCFAs less/g of digesta) (*P* < 0.001). S diet also produced the highest increase in butyric acid concentration with 0.088 μmol more per gram of digesta in comparison to C group (*P* < 0.001). Regarding enzymatic activity in the small intestine, α-glucosidase was influenced by diet (*P* < 0.0001) and by the interaction between diet and cellulose level (*P* = 0.0012) with a higher activity in C and B chickens than in A and S animals (*P* < 0.001). No significant differences were observed in terms of pH, digesta weight and maltase activity (*P* > 0.05).

**Table 3:** Small intestine parameters of broiler chickens described by diet groups

	Diet groups								
General variables	CL	CH	AL	AH	BL	BH	SL	SH	*P*-value
Small intestine weight^1*^	21.5 (1.2)	27.1 (4.3)	29.2 (2.2)	31.1 (2.9)	29.2 (2.3)	29.3 (3.6)	28.7 (1.2)	28.7 (4.2)	< 0.001
Digesta weight^1*^	24.6 (5.6)	19.0 (5.2)	24.1 (6)	26.8 (2.5)	21.4 (3.8)	21.5 (4.4)	24.1 (5.5)	26.2 (3.4)	0.102
Viscosity^2**^	1.9 (1.7–2)	1.7 (1.6–1.8)	2.3 (2–2.6)	2.2 (2.2–2.5)	1.8 (1.6–2)	1.7 (1.6–1.9)	1.6 (1.4–1.8)	1.7 (1.4–2)	0.003
pH^*^	0.9 (0.8–1.0)	1.6 (1.5–1.9)	2.3 (1.9–2.9)	1.9 (1.8–2.2)	2 (1.9–2.2)	2.1 (2.0–2.2)	2.3 (2.2–2.4)	2.7 (2.4–3.2)	0.067
Acetic acid^3*^	0.9 (0.4)	1.7 (0.5)	2.4 (0.6)	2.0 (0.3)	2.1 (0.2)	1.9 (0.7)	2.5 (0.5)	2.8 (0.5)	< 0.001
Butyric acid^3**^	0.03 (0.01–0.03)	0.03 (0.03–0.04)	0.06 (0.05–0.06)	0.09 (0.08–0.09)	0.09 (0.09–0.1)	0.09 (0.07–0.1)	0.1 (0.1–0.12)	0.12 (0.1–0.13)	< 0.001
Putrefactive short chain fatty acid^3**^	0.06 (0.03–0.07)	0.08 (0.03–0.1)	0.06 (0.03–0.09)	0.08 (0.07–0.09)	0.02 (0.02–0.03)	0.01 (0.01–0.02)	0.01 (0.01–0.02)	0.01 (0.00–0.01)	< 0.001
Short chain fatty acid^3*^	1.0 (0.4)	1.9 (0.6)	2.6 (0.7)	2.1 (0.3)	2.2 (0.2)	2.0 (0.8)	2.6 (0.5)	3 (0.5)	< 0.001
α-glucosidase^4*^	7.3 (1.6)	6.4 (2.1)	3.6 (1.9)	4.3 (1.2)	5.0 (0.9)	6.3 (1.1)	4.8 (1.4)	1.4 (0.4)	< 0.001
Maltase^5**^	23.3 (19.9–27.6)	19.5 (14.3–20.0)	25.1 (22.4–28.6)	27 (24.6–27.6)	21.8 (18.8–23.3)	21.9 (17.6–23.1)	25.9 (21.8–27.6)	27 (23.2–28.8)	0.078

*CL* Control diet with 3% of cellulose; *CH* control diet with 6% of cellulose; *AL* 3% inclusion level of apple pomace; *AH* 6% inclusion level of apple pomace; *BL* 3% inclusion level of blackcurrant pomace; *BH* 6% inclusion level of blackcurrant pomace; *SL* 3% inclusion level of strawberry pomace; *SH* 6% inclusion level of strawberry pomace. *SD* standard deviation; IR: interquartile range ^1^ g/kg body weight. ^2^ mPa·s (milliPascal-second). ^3^ μmol/g of digesta.^4^ μmol/h/g (μmol of the product formed per hour per gram of ileal digesta). ^5^ μmol/min/g of protein. *mean (SD), ** median (RI)

**Table 4:** Effect of diet, cellulose level and interaction between them on statistically significant variables

Dependent variables	R-squared	*P*-value			
		Model	Diet	Cellulose level	Diet×Cellulose level
Ileum villus height/crypt depth ratio	0.3171	0.0241	0.0261	0.4811	0.0671
Small intestine weight	0.5009	<0.001	<0.001	0.0340	0.0809
Small intestine viscosity	0.4248	0.0014	<0.001	0.8947	0.5104
Small intestine acetic acid	0.6008	<0.001	<0.001	0.3130	0.0121
Small intestine butyric acid	0.5216	<0.001	<0.001	0.8288	0.8143
Small intestine putrefactive fatty acid short chain	0.3338	0.0161	0.0014	0.9783	0.7377
Small intestine short chain fatty acid	0.5861	<0.001	<0.001	0.3366	0.0154
Small intestine α-glucosidase total activity	0.6390	<0.001	<0.001	0.1815	0.0012
Ceca weight	0.3519	0.0104	0.0048	0.7865	0.1037
Ceca ammonia	0.4699	0.0004	0.0003	0.1003	0.0516
Ceca acetic acid	0.4771	0.0003	0.0013	0.7840	0.0021
Ceca propionic acid	0.5793	<0.001	<0.001	0.1584	0.5162
Ceca isobutyric acid	0.1838	0.2817	0.4120	0.5329	0.1467
Ceca butyric acid	0.5742	<0.001	<0.001	0.7707	0.0048
Ceca isovaleric acid	0.6172	<0.001	<0.001	0.6384	0.0295
Ceca putrefactive fatty acid short chain	0.3856	0.0044	0.0036	0.3433	0.0544
Ceca short fatty acid chain	0.4559	0.0006	0.0023	0.7325	0.0033
Ceca α-glucosidase total activity	0.5195	<0.001	0.0003	0.2658	0.0019
Ceca α-glucosidase release rate	0.3547	0.0097	0.0026	0.0750	0.6231
Ceca α-galactosidase total activity	0.3892	0.0039	0.0051	0.7102	0.0240
Ceca β-galactosidase release rate	0.333	0.016	0.128	0.012	0.087
Ceca β-glucuronidase release rate	0.4048	0.0026	0.0029	0.1564	0.0486
Ceca xilase total activity	0.3123	0.0263	0.0183	0.1712	0.1906
Ceca xilase release rate	0.3877	0.0041	0.0006	0.0632	0.9717

**Table 5:** Linear regression summary for relevant gut parameters

Model	R	R squared	β	*P*-value
Small intestine weight (Y)	0.641	0.411		< 0.001
Apple cellulose diet (β_1_)			5.863	< 0.001
Blackcurrant cellulose diet (β_2_)			4.958	< 0.001
Strawberry cellulose diet (β_3_)			4.387	0.001
Cellulose dose (β_4_)			1.871	0.042
Small intestine viscosity (Y)	0.625	0.391		< 0.001
Apple cellulose diet (β_1_)			0.528	< 0.001
Blackcurrant cellulose diet (β_2_)			−0.055	0.688
Strawberry cellulose diet (β_3_)			−0.96	0.485
Cellulose dose (β_4_)			0.013	0.894
Small intestine short chain fatty acid (Y)	0.682	0.465		< 0.001
Apple cellulose diet (β_1_)			0.946	< 0.001
Blackcurrant cellulose diet (β_2_)			0.657	0.007
Strawberry cellulose diet (β_3_)			1.37	< 0.001
Cellulose dose (β_4_)			0.146	0.380
Small intestine putrefactive short chain fatty acid (Y)	0.559	0.313		0.002
Apple cellulose diet (β_1_)			0.009	0.637
Blackcurrant cellulose diet (β_2_)			−0.057	0.005
Strawberry cellulose diet (β_3_)			−0.052	0.009
Cellulose dose (β_4_)			< 0.001	0.978
Small intestine butyric acid (Y)	0.714	0.51		< 0.001
Apple cellulose diet (β_1_)			0.057	< 0.001
Blackcurrant cellulose diet (β_2_)			0.057	< 0.001
Strawberry cellulose diet (β_3_)			0.088	< 0.001
Cellulose dose (β_4_)			0.002	0.825
Ceca weight (Y)	0.495	0.245		0.015
Apple cellulose diet (β_1_)			0.49	0.028
Blackcurrant cellulose diet (β_2_)			−0.294	0.181
Strawberry cellulose diet (β_3_)			− 0.53	0.806
Cellulose dose (β_4_)			0.040	0.795
Ceca short chain fatty acid (Y)	0.487	0.237		0.018
Apple cellulose diet (β_1_)			2	0.723
Blackcurrant cellulose diet (β_2_)			19.939	0.004
Strawberry cellulose diet (β_3_)			13.31	0.022
Cellulose dose (β_4_)			−1.194	0.765
Ceca putrefactive short chain fatty acid (Y)	0.508	0.258		0.011
Apple cellulose diet (β_1_)			−0.157	0.637
Blackcurrant cellulose diet (β_2_)			−0.963	0.006
Strawberry cellulose diet (β_3_)			0.938	0.007
Cellulose dose (β_4_)			0.211	0.371
Ceca butyric acid (Y)	0.643	0.414		< 0.001
Apple cellulose diet (β_1_)			−0.129	0.934
Blackcurrant cellulose diet (β_2_)			5.987	< 0.001
Strawberry cellulose diet (β_3_)			5.865	< 0.001
Cellulose dose (β_4_)			0.282	0.797

### Cecal parameters

Dietary treatment affected different cecal parameters (Table 6). The effect of diet, cellulose level and interaction between them over the cecal variables are reported in Table 4. In order to explore the magnitude and attitude of the relationship between diet and the evaluated parameters, linear regression results are reported in Table 5. Ce weight was influenced only by diet (*P* = 0.0048). No significant differences were recorded comparing C diets with fruit pomaces diets while a greater increase in the Ce weight was observed in A diet than in B diet (*P* = 0.010). In fact, linear regression showed that replacing C diet with A diet would increase the Ce weight of 0.49 g/kg BW (*P* = 0.027). Moreover, ammonia concentration depended only on diet (*P* < 0.001): a higher ammonia concentration was observed in C group in comparison to B and S groups (*P* = 0.003). As observed in the small intestine, SCFAs and PSCFAs concentration in ceca were influenced by diet (*P =* 0.0023 and *P =* 0.0036, respectively) and interaction between diet and cellulose level (SCFAs only, *P =* 0.0033) with a higher concentration of SCFAs and a lower amount of PSCFAs in B and S diets when compared to C (*P <* 0.001 and P = 0.002, respectively). Particularly, acetic/butyric acid concentration was higher and propionic /isovaleric acid concentration was lower in B and S groups than in C group. Linear regression showed that changing form C to B diet would increase the amount of SCFAs of 16.939 μmol/g of digesta (*P* = 0.004) and decrease PSCFAs of 0.963 μmol/g of digesta (*P* = 0.006) while S diet would increase SCFAs of 13.31 μmol/g of digesta (*P* = 0.021) and decrease PSCFAs of 0.938 μmol/g of digesta (*P* = 0.007). Little differences were found in terms of butyric acid concentration. In fact, changing C diet with B diet would increase it of 5.987 μmol/g of digesta (*P <* 0.001) while S diet would produce an increase of 5.865 μmol/g of digesta (*P <* 0.001).

**Table 6:** Ceca parameters of broiler chickens described by diet groups

	Diet groups								
General Variables	CL	CH	AL	AH	BL	BH	SL	SH	*P*-value
Ceca weight^1*^	3.5 (0.4)	3.6 (0.3)	4.1 (0.5)	3.9 (0.9)	3.4 (0.3)	3.0 (0.4)	3.2 (0.4	3.8 (0.6)	0.01
Digesta weight^1*^	3.2 (0.7)	3.1 (0.7)	3.8 (1.2)	3.6 (1.4)	4.5 (0.9)	4.1 (0.8)	3.3 (0.9)	3.5 (1.2)	0.238
Ammonia^2**^	0.34 (0.27–0.41)	0.38 (0.28–0.44)	0.33 (0.25–0.46)	0.21 (0.19–0.3)	0.27 (0.24–0.3)	0.23 (0.21–0.26)	0.24 (0.19–0.24)	0.22 (0.19–0.28)	0.003
Dry matter^3*^	17.2 (2.1)	16.4 (3.5)	18.8 (3.3)	15.1 (3.4)	16.8 (2.2)	17.9 (3.8)	15.7 (2.6)	17.6 (3)	0.486
pH*	6.5 (0.2)	6.6 (0.2)	6.7 (0.3)	6.5 (0.2)	6.1 (0.3)	6.4 (0.6)	6.4 (0.3)	6.4 (0.32)	0.078
Acetic acid^4*^	78.7 (8.1)	70.7 (11.6)	68.1 (11.6)	87.2 (6.1)	92.2 (7.2)	86.3 (8.8)	90.1 (11.1)	82 (9.7)	< 0.001
Propionic acid^4*^	6.6 (1.8)	5.2 (1.5)	5.4 (1.6)	5.0 (0.6)	3.4 (1.1)	3.0 (0.9)	2.9 (0.7)	2.9 (1.1)	< 0.001
Isobutyric acid^4**^	0.78 (0.37–0.9)	0.72 (0.63–0.77)	0.74 (0.69–0.9)	0.51 (0.33–0.63)	0.51 (0.35–0.59)	0.33 (0.23–0.74)	0.35 (0.27–0.42)	0.39 (0.29–0.63)	0.011
Butyric acid^4*^	16.9 (3.2)	11.1 (3)	11.7 (3.3)	16.1 (3.5)	19 (2.4)	20.9 (3.5)	19.6 (4.3)	20.2 (3.3)	< 0.001
Isovaleric acid^4**^	0.78 (0.52–0.85)	0.87 (0.76–1.1)	0.85 (0.35–0.91)	0.42 (0.31–0.53)	0.42 (0.25–0.58)	0.29 (0.19–0.31)	0.28 (0.2–0.29)	0.25 (0.2–0.37)	< 0.001
Valeric acid^4*^	1.16 (0.33)	0.96 (0.26)	0.93 (0.25)	0.98 (0.24)	0.93 (0.19)	0.81 (0.21)	0.78 (0.23)	0.86 (0.17)	0.204
Putrefactive short chain fatty acid^4**^	2.7 (1.8–3.1)	2.5 (2.3–3.1)	2.5 (2.3–3.1)	1.8 (1.6–2.4)	1.9 (1.5–2.1)	1.5 (1.2–1.8)	1.4 (1.2–1.7)	1.7 (1.5–1.8)	0.002
Short chain fatty acid^4*^	104.7 (7.9)	89.6 (14.5)	88.2 (16.2)	110.2 (9.6)	116.5 (8.3)	111.7 (11.9)	113.9 (12.5)	107 (12.6)	< 0.001

*CL* Control diet with 3% of cellulose; *CH* control diet with 6% of cellulose; *AL* 3% inclusion level of apple pomace; *AH* 6% inclusion level of apple pomace; *BL* 3% inclusion level of blackcurrant pomace; *BH* 6% inclusion level of blackcurrant pomace; *SL* 3% inclusion level of strawberry pomace; *SH* 6% inclusion level of strawberry pomace. *SD* standard deviation; *IR* interquartile range ^1^ g/kg body weight. ^2^ mg/g of protein. ^3^ expressed as percentage. ^4^ μmol/g of digesta. *mean (SD), **median (RI)

Regarding the activity of selected cecal enzymes, α-glucosidase, α-galactosidase total activity, β-galactosidase release rate, β-glucuronidase release rate and xilase activity showed significant differences among dietary treatments (*P* < 0.05, Table 7). In particular, α-glucosidase, α-galactosidase total activity, β-glucuronidase release rate and xilase activity were influenced by diet (*P* < 0.05) and interaction between diet and cellulose level (α-glucosidase, α-galactosidase total activity and β-glucuronidase release rate only, *P* < 0.05). α-glucosidase total activity was lower in A group than in C group being the activity in A group also significantly lower than in B groups. (*P* < 0.0001). On the contrary, α-glucosidase release rate was higher in the fruit pomaces groups (A, B, and S) when compared to C diets (*P* = 0.010). The total activity of α-galactosidase was higher in C group than in A and S groups (*P* = 0.021). In regards to the β-glucuronidase, the release rate of this enzyme was higher in S group when compared to C group being also greater in S group than in B group (*P* = 0.002). No differences were observed for xylase total activity between C group and all the pomaces dietary treatments while the highest activity was found in B group when compared to A group (*P* = 0.026). Xylase release rate was lower in B group than in C, A and S groups (*P* = 0.004). On the contrary, β-galactosidase release rate was influenced by cellulose level (*P* = 0.012), being greater in L diets than in H diets.

**Table 7:** Cecal enzymes activities described by diet groups

	Diet groups								
General Variables	CL	CH	AL	AH	BL	BH	SL	SH	*P*-value
α-glucosidase^1^									
Total activity*	8.0 (1.4)	5.2 (1.1)	3.9 (1.2)	4.7 (0.6)	5.5 (0.8)	6.0 (1.1)	5.6 (1.3)	5.5 (1.5)	< 0.001
Release rate**	65 (56.9–70.8)	75.2 (62.5–91.4)	87.4 (73.2–91.1)	85.5 (84.6–86.2)	87.8 (77.8–93.4)	91.5 (87.4–93.7)	83.9 (75.6–85.6)	85.7 (82.5–87.1)	0.010
α-galactosidase^1^									
Total activity**	24.5 (23.6–32.7)	20.7 (16.9–25.3)	18.3 (10.2–21.5)	20.1 (15.9–23.9)	18.6 (17.5–19.4)	23.5 (22.2–24.6)	16.3 (10.1–18.5)	18.0 (17.2–19.6)	0.021
Release rate**	15.6 (13.4–21.8)	30.6 (17.4–35.2)	26.8 (21.4–37.4)	21.9 (18.9–31.8)	29.9 (24.9–32.9)	25.2 (16.4–27.8)	34.1 (30.6–36.1)	31.6 (28.7–41.7)	0.067
β-galactosidase^1^									
Total activity**	28.8 (22.9–40.9)	19.4 (16.8–24.3)	16.7 (8.9–19)	18.4 (16.4–20.5)	19.6 (14.8–22.7)	21.1 (16.1–28.6)	20.0 (19.8–20.3)	21.6 (16.7–23.1)	0.101
Release rate*	35.7 (13.9)	36.5 (12.8)	53.4 (5.7)	38.9 (8.3)	41.1 (7.5)	40.3 (13.6)	50.2 (9.4)	33.9 (5.6)	0.016
β-glucuronidase^1^									
Total activity*	15.2 (4.9)	15.4 (3.7)	14.2 (2.1)	12.7 (3.6)	11.6 (1.3)	12.0 (4.7)	12.2 (2.2)	13.5 (3.6)	0.416
Release rate*	35.6 (10.2)	45 (9.2)	53.2 (22.4)	41.5 (8.9)	44.3 (12.1)	42.9 (4.1)	65.8 (7.5)	50.4 (6.9)	0.002
Xilase^1^									
Total activity*	5.8 (2.7)	4.8 (1.3)	3.5 (1.3)	4.3 (2.3)	5.3 (1.9)	7.5 (2.3)	4.8 (0.9)	5.6 (1.1)	0.026
Release rate*	38.3 (16.7)	33.7 (18.2)	43.4 (14.9)	34.6 (8.2)	22.8 (10.8)	16.6 (9.8)	46.2 (13.1)	37.4 (5.9)	0.004
α-arabinofuranosidase^1^									
Total activity**	6.0 (4.9–8.7)	5.4 (4.4–5.7)	4.1 (4–5.3)	4.9 (4.6–5.6)	3.9 (2.9–6)	4.8 (3.5–6)	4.4 (3.8–4.8)	4.4 (4.0–5.2)	0.336
Release rate**	69.9 (43.9–72.3)	69.7 (64.1–79.8)	79.4 (76.1–88.2)	76.9 (76.2–81.6)	81 (77.1–82.4))	75.7 (65.9–89.4)	81.8 (76.9–88.1)	88.2 (77.2–91.6)	0.071

*CL* Control diet with 3% of cellulose; *CH* control diet with 6% of cellulose; *AL* 3% inclusion level of apple pomace; *AH* 6% inclusion level of apple pomace; *BL* 3% inclusion level of blackcurrant pomace; *BH* 6% inclusion level of blackcurrant pomace; *SL* 3% inclusion level of strawberry pomace; *SH* 6% inclusion level of strawberry pomace. *SD* standard deviation; *IR* interquartile range ^1^μmol/h/g (μmol of the product formed per hour per gram of protein). *mean (SD), **median (RI)

### Excreta microbiota characterization

A total of 13,872,068 (2 × 250 bp) raw reads were obtained after sequencing and 13,105,029 reads passed the filters applied by QIIME, with a median value of 82,079 (min 2,315 max 251,716) reads/sample and a median sequence length of 465 bp. The rarefaction analysis and the estimated sample coverage indicated that there was a satisfactory coverage of all the samples (ESC median value of 94.23%).

**Fig. 1:**
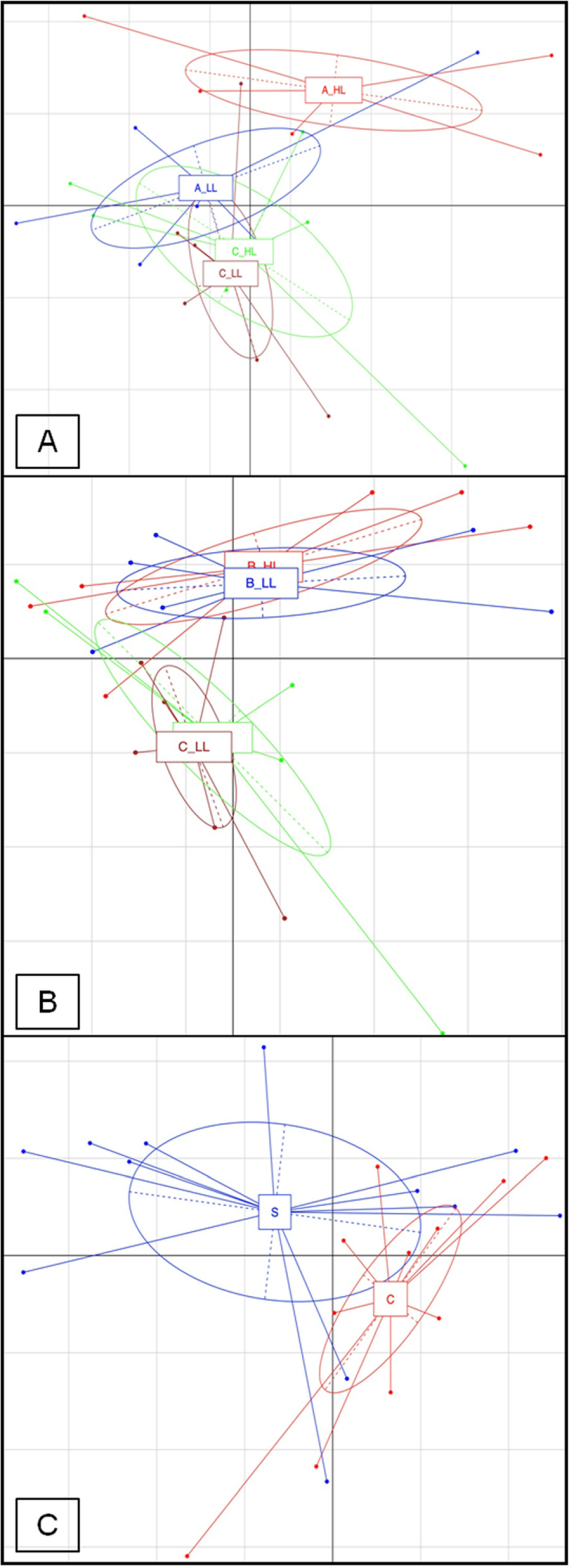
Principal Component Analysis (PCA) based on OTUs relative abundance of broilers with dietary inclusion of fruit pomaces. **a**: apple pomace at high level (A_HL, red) or low level (A-LL, blue) vs. and control cellulose higher level (C_HL, yellow) and control cellulose lower level (C_LL, purple). **b**: blackcurrant pomace at high level (B_HL, red) or low level (B-LL, blue) vs. and control cellulose higher level (C_HL, yellow) and control cellulose lower level (C_LL, purple). **c**: strawberry pomace (S, blue) vs. and control cellulose (C, red)

**Fig. 2:**
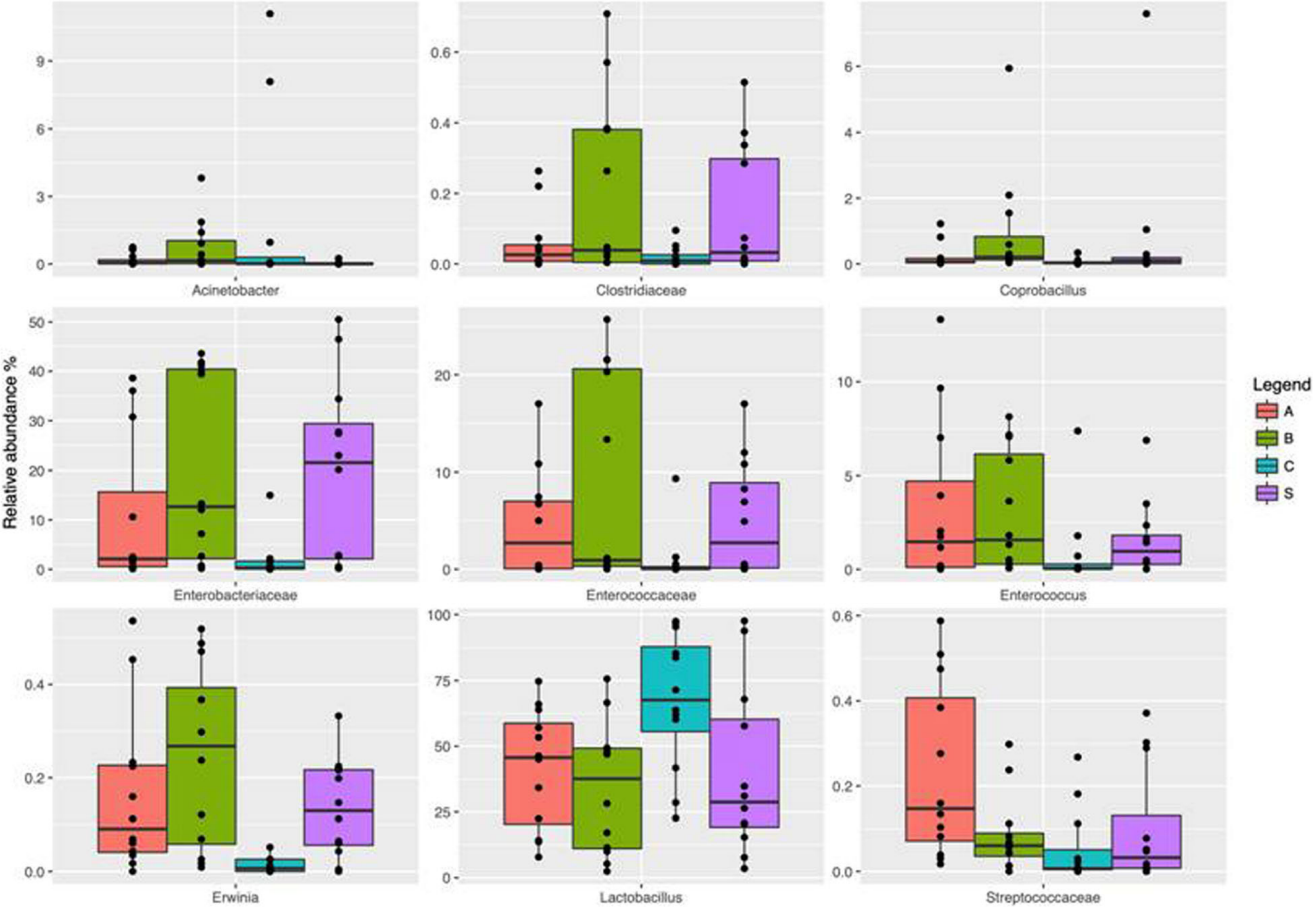
Boxplots showing the relative abundance at genus or family level of the OTUs based on Wilcoxon matched pairs test (*P* ≤ 0.05) in fecal samples of broilers with dietary inclusion of apple (A-red bars), blackcurrant (B-green bars) and strawberry (S-purple bar) compared to control diets (C-blue)

By taking into the account the effects of diet and cellulose level between AH/AL and CH/CL it was possible to observe an increase in the alpha-diversity of the microbiota when comparing AL vs. AH (*P* < 0.05). In regards to the beta-diversity, principal component analysis (PCA) showed a clear shift in the microbiota composition (ADONIS statistical test *P* < 0.05, Fig. 1a). In particular, a clear separation was observed between AH and the rest of the samples, with a significant increase in the abundance of Enterobacteriaceae, Enterococcaceae, Streptococcaceae, *Enterococcus* and *Weissella* (*P* < 0.05), while the relative abundance of *Lactobacillus* genus was higher in CH and lower in AH (*P* < 0.05, Fig. 2).

The utilization of blackcurrant/strawberry pomaces, regardless of cellulose level led to an increase in beta-diversity of excreta microbiota, with a clear separation being observed between B or S diets and C diet (*P* < 0.05, Fig. 1b and c). However, no influence on gut microbiota composition was identified in relation to the alpha-diversity measures (*P* > 0.05). At genus level, the blackcurrant/strawberry groups were characterized by high presence of *Coprobacillus* (B diets), Enterobacteriaceae, Enterococcaceae, *Erwinia* and Erysipelotrichaceae OTUs (FDR < 0.05, Fig. 2).

Due to the little differences observed between the dietary fruit pomace inclusion levels, the authors also compared the fruit pomace-related shift in the gut microbiota independently of their inclusion levels. In regards to the microbiota alpha-diversity, it was possible to observed a reduction in the number of OTUs and rarefaction measures when comparing apple vs. blackcurrant pomaces (*P* < 0.05). On the contrary, an increase in the above-mentioned indices was observed in blackcurrant-fed birds in comparison with strawberry group (*P* < 0.05). Firmicutes, Bacteroides and Proteobacteria were the most abundant phyla in all the groups [see Additional file 4]. However, Firmicutes was differentially abundant among the experimental treatments. In details, the highest and the lowest relative abundance was observed in C and S-fed broilers, respectively (FDR < 0.05). With regards to the most abundant OTUs, both the C- and the fruit pomace-fed groups showed Enterobacteriaceae, *Lactobacillus, L-Ruminococcus* and *Clostridium* as predominant genera in their excreta microbiota [see Additional file 5]. Comparing the relative abundance of the main OTUs across the samples Enterobacteriaceae, Enterococcaceae, Streptococcaceae, *Enterococcus* and *Erwinia* OTUs were enriched by fruit pomace utilization, while a significant reduction in *Lactobacillus* was observed (*P* < 0.05).

## Discussion

The present study evaluated the effects of dietary fruit pomace inclusion on growth performance, intestinal morphology, microbiota, small intestine and cecal indices of broiler chickens. The fibre content analysis of dried fruit pomaces showed relatively small differences in the content of insoluble dietary fibre fraction, whereas high differences were detected in terms of soluble fraction (SDF). The highest content of SDF was attributed to the apple preparation (9.10%), while the strawberry one contained almost no soluble fibre (0.40%). This in accordance with what has previously been found in other studies [9, 10, 34]. The most important bioactive components in fresh fruit and subsequently in dried fruit pomaces are polyphenolic compounds [9, 35]. In the present trial, the total polyphenol concentration in apple preparation was the lowest (below 9 mg/g), but it was more than three times higher in blackcurrant and strawberry pomaces. Very comparable polyphenol contents were observed by Juśkiewicz et al. [14, 36] in previous studies conducted on growing turkeys. As a result of dietary pomace inclusion, the polyphenol concentration increased from 4 to 29 and from 2 to 17 mg/g in the starter and grower diets, respectively.

Growth performances were not affected by dietary fruit pomace inclusion (*P* < 0.05). Similar findings have been reported in young turkeys fed 5% of dried apple, blackcurrant and strawberry pomaces [10]. However, it is important to state that all the chickens regardless of dietary treatments showed a BW at 35 day of 1.9–2 kg which is lower in comparison to standard growth performance for Ross 308 [37] This can be due to the fact that the dietary treatments fed to the animal of the present study are less concentrate in terms of energy and crude protein than the commercial standard diets for broilers [38].

Dietary fruit pomace inclusion did not affect the morphometric indices of the broiler chickens. It is well known that the physiological gut development is characterized by long villi and shallow crypts: longer villi are associated with increased absorption of nutrient [24], while shallower crypts reflect a prolonged survival of villi without the need of renewal [39]. On the contrary, lower villus height and greater crypts depth are associated with poor digestion, less absorption of nutrient and poor growth performances [25]. Since both the intestinal morphology and the growth performance of the fruit pomace fed broilers in the current research were unaffected, it is reasonable to hypothesize that fruit pomace meal utilization does not negatively influence gut development or nutrient absorption. Apart from dietary treatments, morphometric indices showed a proximodistal decreasing gradient from duodenum to ileum. This finding is in accordance with the available literature [32, 40] and with the physiological development of the absorption processes. Indeed, the duodenum is the intestinal segment with the fastest cell renewal and the first gut segment to receive the physical, chemical and hormonal stimuli caused by the presence of the diet in the lumen [41]. Furthermore, the jejunum is an important site for nutrient digestion [42].

It is well known that the dietary content and physicochemical properties of different fibre fractions may provoke physiological changes in the small intestine, and subsequently in the lower gut [27, 43]. More soluble fibre as dietary ingredient may considerably affect intestinal viscosity, transit time, digesta moisture, and other indices of intestinal function [44]. In the present study, dietary fruit pomace inclusion significantly increased the SI relative mass when compared to C diets while Ce weight seems to increase in A diet when compared to the other dietary treatments. Addition of fiber to diets largely results in enlargement of digestive tissues, presumably due to increase retention time and digestion of the diet. In fact, Savory and Gentle [45], Kehoe et al. [46], and Williamson et al. [47] found an increase in intestinal relative weight in quails and mallard fed different fiber sources. In the present study, the ileal viscosity was also affected by dietary treatment. In particular, A group showed the highest ileal viscosity. This effect could be partly ascribed to the SDF content and to the different concentrations of non-starch polysaccharides (NSP). As reported in literature, the apple pomace excelled the blackcurrant and strawberry pomaces not only in the content of water-soluble NSPs, but also in the content of NSP monomers such as arabinose, galactose and uronic acid [36]. However, in the present study all the observed viscosity levels were below those that may provoke some undesired physiological effects. Indeed, high digesta viscosity (ranging from 4 to 5 mPa·s) may not only constrain absorption of nutrients, but also be a stimulus for the overgrowth of microbiota and enhanced putrefactive processes in the lower small intestine [48]. Furthermore, a significant decrease in the activity of α-glucosidase in the small intestine was noticed in A and S animals compared to C and B groups with no variations being detected in the activity of maltase. It has been reported that consumption of high levels of polyphenols may effectively diminish the activity of brush border enzymes in the small intestine [49, 50], thus potentially resulting into less-effective carbohydrate digestion. Indeed, birds fed high-fiber diets had significantly depressed mass-specific small intestinal sucrase activities when compared to birds fed low-fiber diets [51]. Fiber seemed to decrease mass-specific activities of small intestinal sucrase also in geese [51]. This is in contrast with what has previously been demonstrated in chickens fed mannan oligosaccharides, which increased disaccharidase activity [42]. A recent study on growing turkeys fed diets containing 5% of apple, blackcurrant or strawberry pomaces showed significant decrease in sucrase and/or maltase mucosal activity in blackcurrant and strawberry groups, but not in the apple group [36]. These conflicting results could be related to the different fiber components (e.g., pectin vs. cellulose), which can have differential effects on enzyme activities [52]. Also in the ceca, modulation of digestive enzyme activities is one of the mechanism by which the gastrointestinal tract can respond to changes in food composition and quality [51].

In this study, the fruit pomace groups showed lower cecal total activity of α-glucosidase and α-galactosidase than C group. Regarding enzymes release rate, α-glucosidase release rate was higher in the fruit pomaces groups (A, B, and S) when compared to C diet while β-galactosidase release rate was higher in L diets than in H diets.

Very few studies have measured the activities of exogenous (in that case bacterial) digestive enzymes in avian cecal digesta, and some of these variations are still difficult to explain. It should be stressed that the release rate of β-glucuronidase was lower in C, B and A groups when compared to S group and it can be considered a positive finding as this enzyme is characteristic for the harmful bacteria species due to its deconjugative properties that support the transformation of xenobiotics into more toxic substances [53]. Moreover, the increase of α-glucosidase release rate into the cecal environment of birds fed fruit pomace diets could be probably due to the higher content of flavonoid glycosides. Importantly, flavonoid glycosides can undergo bacterial deglycosylation in the hindgut, and the released aglycones are furtherly transformed into a smaller number of metabolites such as phenolic acids and phloroglucinol, which also contribute to the health benefits of polyphenol consumption [54].

Ileal SCFAs were also higher in the pomace groups than in C diet (*P* < 0.05). The SCFAs have positive health effects, especially butyric acid, which has been shown to have anti-inflammatory properties, to modulate oxidative stress and to be a main energy substrate for enterocytes [55, 56]. Similar effects have been ascribed to propionic acid, but generally to a lesser extent, while acetic acid is associated with fewer physiological effects [57]. On the contrary, ileal PSCFAs concentrations were lower in S and B diets when compared to A and C groups. This is in accordance with the literature as the polyphenols were reported to significantly reduce the anaerobic bacterial fermentation of polypeptides and amino acids [58]. In ceca, dietary S and B inclusion also influenced the ammonia concentration. In particular, B and S diets showed lower ammonia concentration when compared to C groups (*P* < 0.05). The decreased ammonia concentration could be related to the higher ratio of the ETs to flavan-3-ols and could be considered a positive change since this compound can produce cell necrosis, alter nucleic acid synthesis, induce cancerous cell growth and increase viral infections in the lower bowel [13, 26].

Dietary fruit pomace inclusion also determined a modification of the gut microbiota composition in broilers. Investigating the differences between broiler chickens fed the control and the fruit pomaces-based diets in the present study, no differences were found in regards to α-diversity measures. Comparing the different fruit pomaces, an increase of α-diversity was observed when comparing AH vs. AL and B vs. S diet, while a decrease was observed comparing A and B diets. Concerning β-diversity, a clear separation of excreta microbiota due to dietary fruit pomaces inclusion was, however, observed. High levels of diversity generally help intestinal microbiota to maintain stability after environmental stress, as well as to determine effective colonization resistance against potential invading pathogens [59]. For these reasons, a reduction of alpha and beta diversity should be considered negative. The majority of the studies reported that the most common phyla in the chicken microbiota are Firmicutes, Bacteroides and Proteobacteria [60] and this in accordance with the findings of the present study. However, Firmicutes was higher in the C group and lower in the S group. Firmicutes phylum is known to be involved in the breakdown of otherwise indigestible polysaccharides such as resistant starch and cellulose [61, 62]. Therefore, the observed differences in microbial composition may influence the food to energy conversion capacity. Moreover, the presence of Firmicutes is positively associated with the production of acetate, which can have a beneficial effect on gut health [63]. At genus level, *Clostridium*, *Ruminococcus*, *Lactobacillus* and *Bacteroides* [64] have been reported to be the most abundant genera in the chicken microbiota. In this study, a shift in the relative abundance of the main taxa with an increase of Enterobacteriaceae and Enterococcaceae families was observed in all the fruit pomace groups. In particular, an increase of *Weissella* in AH and *Erwinia* in S/B diets, as well as a decrease of *Lactobacillus*, were detected in broilers fed pomace-based diets. The shift recorded in the present trial could represent a potential negative modulation, since several poultry pathogens (such as *E. coli* and *Salmonella* spp.) are members of Enterobacteriaceae family. However, the genus *Erwinia (*Enterobacteriaceaea family*)* seems to be a common commensal bacteria in broilers microbiota [65]. Enterococcaceae are also well-known to have intrinsic resistance to some antibiotics (i.e., cephalosporins), thus potentially worsening the antimicrobial resistance issues in poultry [66]. Furthermore, *Weissella* is a member of the lactic acid bacteria (LAB) that can be normally isolated from the gastrointestinal tract of animals [67]. Several *Weissella* species seemed to be able to produce significant amounts of non-digestible oligosaccharides and extracellular polysaccharides that could act as prebiotics. Several *Weissella* strains have also been found to act as probiotics, mainly due to their antimicrobial activity (i.e bacteriocinogenic strains) [67], thus representing a positive pomace-related modulation.

In regards to the *Lactobacillus* genus, it is well known that this typical probiotic bacterium promotes the homeostasis of immune cells and the intestinal health [68]. Furthermore, Lactobacilli are considered to play an important regulatory role in the gut by producing large amounts of lactic acid and lactate that can be converted to SCFAs and lead to a pH reduction [68]. They also seemed to be able to produce bacteriocins that create an unfavorable environment for pathogens such as enterobacteria in the anterior part of the gastrointestinal tract, thus potentially protecting the host from disease development [69]. For these reasons, a reduction of the *Lactobacillus* genus could represent a negative modulation by fruit pomace utilization.

## Conclusion

In conclusion, the results of the present study suggest that fruit pomaces could be a new, low-cost fiber source in poultry nutrition. Indeed, they did not impair growth performance or gut morphometry/histopathology, they improved the production of SCFAs and they reduce the production of PSCFAs both in the small intestine and in the ceca. However, they showed a negative effect by increasing SI and ceca weight and SI digesta viscosity. A potential negative modulation of gut microbiota with a decrease of *Lactobacillus* spp. and an increase of Enterobacteriaceae and Enterococcaceae has also been observed. As changing C diet with A diet seems to provoke a higher increase of SI weight or viscosity without decreasing the production of PSCFA in SI and ceca when comparing to B and S diet, B and S seems to be the most promising fiber sources. The present study had several limitations concerning low statistical power due to the small sample size and further studies with a higher number of animals seem necessary to better understand which doses and which fruit pomaces should be more suitable for poultry nutrition.

## Supplementary information

**Additional file 1.** Chemical composition, including polyphenolic fraction in dried fruit pomaces.

**Additional file 2. ** Nutritional value of diets fed to broilers at starter and grower period.

**Additional file 3.** Effects of fruit pomace inclusion on growth performances of broilers fed experimental diets.

**Additional file 4.** Raw data of phyla detected in broiler chickens fed experimental diets.

**Additional file 5.** Raw data of genera detected in broiler chickens fed experimental diets.

## Data Availability

The datasets analyzed in the present study are available from the corresponding author on reasonable request.

## Abbreviations

A: Apple
AL: 3% inclusion level of apple pomace
AH: 6% inclusion level of apple pomace
B: Blackcurrant
BL: 3% inclusion level of blackcurrant pomace
BH: 6% inclusion level of blackcurrant pomace
BW: Body weight
BWG: Body weight gain
C: Control
Cd: Crypt depth
Ce: Ceca
CL: Control diet with 3% of cellulose
CH: Control diet with 6% of cellulose
DFI: Daily feed intake
DM: Dry matter
FCR: Feed conversion ratio
FDR: False discovery rate
GALT: Gut-Associated Lymphoid Tissue
GC: Gas cromatography
H: High cellulose
HE: Haematoxilyn and eosin
HPLC DAD: High-performance liquid chromatography diode array detector
IDF: Insoluble dietary fibre
L: Low cellulose
OTU: Operational taxonomic unit
PCA: Principal component analysis
PSCFA: Putrefactive short chain fatty acids
S: Strawberry
SCFAs: Short-chain fatty acids
SDF: Soluble dietary fibre
SEM: Standard error of the mean
SH: 6% inclusion level of strawberry pomace
SI: Small intestine
SL: 3% inclusion level of strawberry pomace;
TDF: Total dietary fibre
Vh: Villus height
Vh/Cd: Villus height to crypt depth
