# Supplementary material for: Dried fruit pomace inclusion in poultry diet: growth performance, intestinal morphology and physiology
Source: J Anim Sci Biotechnol. 2020 Jun 19;11:63. doi: 10.1186/s40104-020-00464-z (PMC7304194; doi:10.1186/s40104-020-00464-z)
Supplement: Supplementary file 1 — Additional file 1. Chemical composition, including polyphenolic fraction in dried fruit pomaces. [file 40104_2020_464_MOESM1_ESM.docx]

**Additional file 1. Chemical composition, including polyphenolic fraction in dried fruit pomaces**

|  | Apple pomace | Blackcurrant pomace | Strawberry pomace |
| --- | --- | --- | --- |
| Dry matter, % | 92.4 | 93.7 | 93.2 |
| Crude ash, % | 1.10 | 3.89 | 8.01 |
| Crude protein, % | 6.64 | 15.5 | 16.4 |
| Crude fat, % | 2.63 | 13.8 | 10.4 |
| Crude fiber, % | 22.0 | 19.8 | 31.4 |
| Macroelements, % |  |  |  |
| Calcium (Ca) | 0.09 | 0.36 | 0.37 |
| Potassium (K) | 0.23 | 0.39 | 0.15 |
| Phosphorus (P) | 0.16 | 0.33 | 0.43 |
| Magnesium (Mg) | 0.04 | 0.17 | 0.10 |
| Sodium (Na) | <0.01 | <0.01 | <0.01 |
| Fibre, % |  |  |  |
| TDF | 60.8 | 60.6 | 52.8 |
| IDF | 51.7 | 52.9 | 52.5 |
| SDF | 9.10 | 7.70 | 0.40 |
| Polyphenols, mg/g |  |  |  |
| Total polyphenols HPLC | 8.43 | 26.7 | 28.9 |
| Anthocyanins | 0.00 | 3.74 | 0.14 |
| Chlorogenic acid | 0.26 | 0.00 | 0.00 |
| Ellagic acid | 0.00 | 0.00 | 0.57 |
| Ellagitannins | 0.00 | 0.00 | 11.2 |
| Agrimoniin | 0.00 | 0.00 | 4.19 |
| Myricetin glycosides | 0.00 | 0.34 | 0.00 |
| Kaempferol glycosides | 0.00 | 0.00 | 0.04 |
| Kaempferol | 0.00 | 0.03 | 0.06 |
| Quercetin glycosides | 0.83 | 0.00 | 0.27 |
| Quercetin | 0.03 | 0.06 | 0.00 |
| Phloridzin | 0.55 | 0.00 | 0.00 |
| Tiliroside | 0.00 | 0.00 | 0.85 |
| Flavan-3-ols | 6.76 | 22.5 | 15.8 |
| Procyanidins | 6.75 | 22.5 | 15.8 |
| Free catechins | 0.02 | 0.01 | 0.03 |

TDF: Total dietary fibre; IDF: Insoluble dietary fibre; SDF: soluble dietary fibre
