# Supplementary material for: Dried fruit pomace inclusion in poultry diet: growth performance, intestinal morphology and physiology
Source: J Anim Sci Biotechnol. 2020 Jun 19;11:63. doi: 10.1186/s40104-020-00464-z (PMC7304194; doi:10.1186/s40104-020-00464-z)
Supplement: Supplementary file 2 — Additional file 2. Nutritional value of diets fed to broilers at starter and grower period. [file 40104_2020_464_MOESM2_ESM.docx]

**Additional file 2. Nutritional value of diets^*^ fed to broilers at starter and grower period.**

|  | Starter diets (days 1-14) | | | | | | | |  | Grower diets (days 15-35) | | | | | | | |
| --- | --- | --- | --- | --- | --- | --- | --- | --- | --- | --- | --- | --- | --- | --- | --- | --- | --- |
|  | CL | CH | AL | AH | BL | BH | SL | SH |  | CL | CH | AL | AH | BL | BH | SL | SH |
| Dry matter^1^, % | 87.7 | 87.0 | 85.9 | 83.5 | 85.9 | 83.4 | 85.9 | 83.4 |  | 87.8 | 87.1 | 86.0 | 83.6 | 86.0 | 83.4 | 86.0 | 83.5 |
| Crude protein^1^, % | 21.5 | 21.5 | 21.5 | 21.5 | 21.5 | 21.5 | 21.5 | 21.5 |  | 19.5 | 19.5 | 19.5 | 19.5 | 19.5 | 19.5 | 19.5 | 19.5 |
| ME^1^, kcal | 2950 | 2950 | 2950 | 2950 | 2950 | 2950 | 2950 | 2950 |  | 3100 | 3100 | 3100 | 3100 | 3100 | 3100 | 3100 | 3100 |
| Crude fat^1^, % | 4.80 | 4.80 | 5.97 | 7.17 | 5.89 | 6.97 | 5.83 | 6.86 |  | 6.46 | 6.50 | 7.63 | 8.81 | 7.55 | 8.64 | 7.49 | 8.52 |
| Crude fibre^1^, % | 3.33 | 3.98 | 3.23 | 3.98 | 3.32 | 3.98 | 3.32 | 3.97 |  | 3.23 | 3.88 | 3.23 | 3.88 | 3.22 | 3.88 | 3.22 | 3.87 |
| Lysine^1^, % | 1.34 | 1.34 | 1.34 | 1.34 | 1.34 | 1.34 | 1.34 | 1.34 |  | 1.18 | 1.18 | 1.18 | 1.18 | 1.18 | 1.18 | 1.18 | 1.18 |
| Methionine (Met)^1^, % | 0.73 | 0.73 | 0.73 | 0.73 | 0.73 | 0.73 | 0.73 | 0.73 |  | 0.62 | 0.62 | 0.62 | 0.62 | 0.62 | 0.62 | 0.62 | 0.62 |
| Met + Cystine^1^, % | 1.11 | 1.11 | 1.11 | 1.11 | 1.11 | 1.11 | 1.11 | 1.11 |  | 0.97 | 0.97 | 0.97 | 0.97 | 0.97 | 0.97 | 0.97 | 0.97 |
| Threonine^1^, % | 0.92 | 0.92 | 0.92 | 0.92 | 0.92 | 0.92 | 0.92 | 0.92 |  | 0.83 | 0.83 | 0.83 | 0.83 | 0.83 | 0.83 | 0.83 | 0.83 |
| Calcium^1^, % | 1.01 | 1.01 | 1.01 | 1.01 | 1.01 | 1.01 | 1.01 | 1.01 |  | 0.85 | 0.85 | 0.85 | 0.85 | 0.85 | 0.85 | 0.85 | 0.85 |
| Phosphorus available^1^, % | 0.38 | 0.38 | 0.38 | 0.38 | 0.38 | 0.38 | 0.38 | 0.38 |  | 0.42 | 0.42 | 0.42 | 0.42 | 0.42 | 0.42 | 0.42 | 0.42 |
| Sodium^1^, % | 0.17 | 0.17 | 0.17 | 0.17 | 0.17 | 0.17 | 0.17 | 0.17 |  | 0.15 | 0.15 | 0.15 | 0.15 | 0.15 | 0.15 | 0.15 | 0.15 |
| Fibre fraction^2^, % |  |  |  |  |  |  |  |  |  |  |  |  |  |  |  |  |  |
| TDF | 14.8 | 15.0 | 14.4 | 14.9 | 12.8 | 13.8 | 12.8 | 15.1 |  | 12.8 | 15.3 | 17.7 | 17.5 | 13.1 | 15.1 | 13.8 | 14.4 |
| IDF | 12.7 | 13.3 | 13.3 | 13.8 | 12.3 | 13.0 | 12.2 | 13.2 |  | 12.4 | 14.3 | 14.7 | 15.4 | 11.8 | 13.1 | 13.0 | 13.9 |
| SDF | 2.00 | 1.70 | 1.00 | 1.10 | 0.60 | 0.70 | 0.60 | 1.90 |  | 0.40 | 1.00 | 3.00 | 2.10 | 1.20 | 2.00 | 0.70 | 0.50 |
| Polyphenols^2^, mg/g |  |  |  |  |  |  |  |  |  |  |  |  |  |  |  |  |  |
| Total | 0.91 | 0.98 | 0.95 | 1.06 | 0.97 | 1.17 | 1.00 | 1.27 |  | 0.81 | 0.88 | 0.83 | 1.00 | 0.84 | 1.04 | 0.91 | 1.05 |
| Flavan-3-ols | 0.18 | 0.18 | 0.27 | 0.41 | 0.86 | 1.37 | 0.61 | 1.05 |  | 0.15 | 0.17 | 0.35 | 0.47 | 0.61 | 1.69 | 0.45 | 0.94 |
| Procyanidins | 0.18 | 0.18 | 0.27 | 0.41 | 0.86 | 1.37 | 0.61 | 1.05 |  | 0.14 | 0.17 | 0.34 | 0.47 | 0.61 | 1.69 | 0.44 | 0.94 |
| Free catechins | trace | trace | trace | trace | trace | trace | trace | trace |  | trace | trace | trace | trace | trace | trace | trace | trace |

^1^ the content of the nutrients and non-nutrients was calculated according to the Polish Feedstuff Analysis Tables (Smulikowska and Rutkowski [20]) and the analysed fruit pomaces (see Material & Methods section). ^2^analysed content (see Material & Methods section).CL: Control diet with 3% of cellulose; CH: control diet with 6% of cellulose; AL: 3% inclusion level of apple pomace; AH: 6% inclusion level of apple pomace; BL: 3% inclusion level of blackcurrant pomace; BH: 6% inclusion level of blackcurrant pomace; SL: 3% inclusion level of strawberry pomace; SH: 6% inclusion level of strawberry pomace; ME: metabolizable energy; TDF: Total dietary fibre; IDF: Insoluble dietary fibre; SDF: soluble dietary fibre.
