# Supplementary material for: Dried fruit pomace inclusion in poultry diet: growth performance, intestinal morphology and physiology
Source: J Anim Sci Biotechnol. 2020 Jun 19;11:63. doi: 10.1186/s40104-020-00464-z (PMC7304194; doi:10.1186/s40104-020-00464-z)
Supplement: Supplementary file 3 — Additional file 3. Effects of fruit pomace inclusion on growth performances of broilers fed experimental diets. [file 40104_2020_464_MOESM3_ESM.docx]

**Additional file 3. Effects of fruit pomace inclusion on growth performances of broilers fed experimental diets**

|  | Diet groups | | | | | | | |  |
| --- | --- | --- | --- | --- | --- | --- | --- | --- | --- |
| General variables | CL | CH | AL | AH | BL | BH | SL | SH | *P*-value |
| BW^1^ |  |  |  |  |  |  |  |  |  |
| Day 14* | 0.39 (0.01) | 0.4 (0.02) | 0.4 (0.3) | 0.39 (0.01) | 0.4 (0.02) | 0.4 (0.02) | 0.4 (0.02) | 0.4 (0.02) | 0.787 |
| Day 35* | 1.9 (0.09) | 2 (0.07) | 2 (0.1) | 2 (0.09) | 2 (0.09) | 2 (0.08) | 2 (0.08) | 2 (0.03) | 0.792 |
| DBWG^2^ |  |  |  |  |  |  |  |  |  |
| Day 1-14* | 24.7 (0.7) | 25.1 (1.3) | 25.3 (1.9) | 24.2 (1.1) | 24.9 (1.5) | 24.9 (1.6) | 25.3 (1) | 25.4 (1.1) | 0.771 |
| Day 15-35* | 73.2 (4.1) | 75.5 (1.9) | 74.5 (4.2) | 73.9 (4.5) | 76.4 (3.6) | 73.3 (3) | 74.1 (2.8) | 74.5 (1.6) | 0.747 |
| Day 1-35* | 53.5 (2.9) | 55.3 (1.3) | 54.5 (3.1) | 53.6 (2.8) | 55.5 (2.3) | 53.6 (2.4) | 54.3 (1.9) | 54.8 (0.9) | 0.700 |
| FCR day 1-35^3^** | 1.65  (1.6-1.75) | 1.64  (1.61-1.65) | 1.64  (1.57-1.66) | 1.67  (1.66-1.72) | 1.65  (1.63-1.7) | 1.7  (1.67-1.81) | 1.68  (1.66-1.79) | 1.68  (1.61-1.69) | 0.194 |
| DFI day 1-35^2^* | 89.8 (3.3) | 90.5 (1.5) | 89.2 (2.5) | 90.6 (3.3) | 92.3 (1.9) | 92.5 (2.7) | 93.3 (2.2) | 91.5 (1.9) | 0.078 |

CL: Control diet with 3% of cellulose; CH: control diet with 6% of cellulose; AL: 3% inclusion level of apple pomace; AH: 6% inclusion level of apple pomace; BL: 3% inclusion level of blackcurrant pomace; BH: 6% inclusion level of blackcurrant pomace; SL: 3% inclusion level of strawberry pomace; SH: 6% inclusion level of strawberry pomace. SD:standard deviation; IR: interquartile range. ^1^ Expressed in kg. ^2^ Expressed in gr. ^3^ Expressed in kg/ kg. BW: body weight; DBWG: daily body weight gain; DFI: daily feed intake; FCR: feed conversion ratio. * mean (SD), ** median (RI)
